# Controllable Preparation of Eucommia Wood-Derived Mesoporous Activated Carbon as Electrode Materials for Supercapacitors

**DOI:** 10.3390/polym15030663

**Published:** 2023-01-28

**Authors:** Hongyu Su, Caining Lan, Zhouping Wang, Lin Zhu, Mingqiang Zhu

**Affiliations:** 1 College of Mechanical and Electronic Engineering, Northwest A&F University, Yangling 712100, China; 2 Northwest Research Center of Rural Renewable Energy Exploitation and Utilization of M.O.A, Northwest A&F University, Yangling 712100, China; 3 Horticultural Technology Station of Shaanxi, Xi’an 710003, China

**Keywords:** Eucommia ulmoides Oliver, systemic activation processes, mesoporous-activated carbon, economic efficiency assessment, supercapacitor

## Abstract

Activated carbons (ACs) for supercapacitors were synthesized from Eucommia ulmoides Oliver (EUO) wood by H_3_PO_4_ with systemic activation processes. The target structure of ACs could be prepared by adjusting the technological parameters. As the H_3_PO_4_ concentration was 25%, the mass ratio of feedstocks to activator was 1:4, the activation time was 6 h, and the activation temperature was 400 °C, the obtained AC revealed a high specific surface area (2033.87 m^2^·g^−1^) and well-developed mesoporous (the rate of mesoporous was 96.4%) with the best economic feasibility. Besides, it possessed excellent electrochemical performance: the maximum specific capacitance reached up to 252 F·g^−1^, the charging and discharging period was 3098.2 s at 0.2 A·g^−1^, and the retention rate of specific capacitance reached 92.3% after 10,000 cycles. This low temperature and convenience technology provide a valuable reference for synthesizing the EUO-based ACs, making high-value utilization on the EUO branches, and owning a broad application prospect in supercapacitors.

## 1. Introduction

With the consumption of fossil fuels and other non-renewable energy sources, the research of energy storage technology has become the focus of scientific and industrial areas [1]. Supercapacitors are one of the most efficient energy storage devices, which are widely applied in electronics, electric cars and military equipment because of their high specific capacitance, energy density, fast charge–discharge property and durability [2].

Generally, supercapacitors are divided into electrochemical double-layer capacitors (EDLCs) and pseudo-capacitors (PCs) based on the mechanism of charge storage. The frequent-used electrodes for PCs are metal oxides and conductive polymers [3]. Although the redox reaction on the electrode can provide a huge specific capacitance, the poor cyclic stability and high price limit its practical application. On the contrary, in the field of EDLCs, carbon-based electrodes could demonstrate satisfactory stability and charge transfer rate, which is attributed to the accumulation of the pure electrostatic charge at the interface between the electrolyte and electrode. According to previous research, various kinds of carbon molecular structures have been obtained via artificial means, such as graphene [4], carbon nanotubes [5], carbon fiber [6], carbon aerogel [7] and activated carbon (AC) [8]. Among them, AC was more suitable to scientific research and commercial applications because of the high specific surface area, well-developed pore structures, simple preparation, low cost and durability. Given the awareness of the sustainable development and the energy crisis, the regenerative and sustainable feedstock to replace the traditional one for electrode materials is becoming increasingly urgent. Biomass renewables were a satisfactory precursor, which has been widely used for the synthesis of ACs due to the simple acquisition, convenient preparation, environment friendly and low cost, such as coconut shell [9], liquor waste [10], crab shell [11], silkworm cocoon [12], corncob [13], dead leaf [14], etc. Moreover, ACs can be prepared by physical or chemical activation methods. In the physical activation methods, oxidizing gases (water vapor, CO_2_, and O_2_) are used as activators. The ACs’ products are mainly granular with microporous structures because the carbon atoms in raw materials are oxidized into gas products such as CO and CO_2_, leaving nano-level pores inside. In the chemical activation method, the solid or liquid chemical activators (ZnCl_2_, phosphoric acid, calcium chloride, and sodium hydroxide) were applied to mix with the precursor, which can reduce the activation temperature in the preparation of ACs [15]. The chemical activation has a shorter reaction time and lower reaction temperature than physical activation, producing activated biochar with a higher SSA and total pore volume [16]. During the pyrolysis process, the feedstock was transformed into carbides containing activators and their derivatives through a chemical reaction; numerous pores were generated after washing out these impurities [17]. Usually, the H_3_PO_4_ activator is suitable for the cell wall structure of the wood and other plant fiber materials. The main mechanism of activation is that it can form a phosphoric acid-biopolymer complex during the hydrolysis process of H_3_PO_4_, which has a significant effect on the structure of the AC products [18]. Recently, the preparation of mesoporous AC activated by H_3_PO_4_ was well developed and was promising in the applications of supercapacitors [19].

The primary defect of biomass carbon-derived electrode materials was the low specific capacitance, and the primary factors affecting the magnitude of the specific capacitance were the surface and internal structure of carbon. Therefore, many researchers have given a lot of attention to exploring carbon material with specific surface area, pore distribution, material resistance, and surface modification to enhance the electrochemical performance. Wang et al. [20] synthesized the rotten potatoes-based-ACs by different activation conditions. When the feedstock was treated at 900 °C for 3 h, the specific surface area of the obtained AC with a hierarchical pore structure reached 2201 m^2^·g^−1^, and the proportion of mesoporous was 67.63%, which worked well in supercapacitors. Moreover, the synthesized ACs prepared with the Syzygium Oleanna leaf, appeared nanosheet-modified by nanofibers when the activation temperature was between 500 °C and 600 °C, while the high-density nanosheets with a bloom shape were prepared at the activation temperature of 700 °C. The different morphologies demonstrated the different pore size structures and specific surface areas in ACs, which further exhibited the difference in the electrochemical performance in the two electrode systems (the maximum specific capacitance was 188 F·g^−1^; the minimum specific capacitance was 114 F·g^−1^) [21]. Wan et al. utilized MgCO_3_ as an activator, and a series of ACs were produced with different feedstock-activator ratios. The results demonstrated that the pine pollen based-hierarchical porous carbon (high ratios of meso-/macropore) synthesized with a small quantity of MgCO_3_, as the activator possessed a high content of N (3.13 at.%), O (14.71 at.%), and high capacitance (419.6 F·g^−1^ at 1 A·g^−1^), which illustrated that the different proportions of activators affect the pore structure and the surface functional groups directly [22]. The concentration of the activator [23], activation temperature, activation time, and the mass ratio of feedstocks to activator play vital roles in the formation of ACs’ pores structure and functional groups, which were the primary influencing factors of the ACs applied in the supercapacitor. It was possible to obtain the expected ACs by changing these processes of activation. However, the few types of research analyze these technological parameters systematically; furthermore, the whole economic efficiency assessment work was also rarely explored.

Eucommia ulmoides Oliver (EUO) is a kind of traditional Chinese medicine that is abundant in China. The leaf-forest model of EUO can harvest 45 tons of products per hectare (22.5 tons of branches, 15 tons of leaves, and 7.5 tons of bark) with about 156,750 CNY, while the value of branches is only 6750 CNY. Our previous studies have indicated that the EUO wood could be employed in preparation of activated carbon for supercapacitors with high performance [24,25]. In this study, phosphoric acid was used as an activator to explore the optimal production process, which facilitated the industrial production of AC from EUO wood. The influence of reaction parameters such as activation temperature, activation time, and phosphoric acid concentration on the specific surface area, surface morphology, and electrochemistry, and other properties of the produced AC was systematically investigated. Moreover, the costs of different processes were also estimated. This report can provide some theoretical references for the research and production of EUO wood-based AC electrode materials.

## 2. Experimental Section

### 2.1. Preparation of EUO Wood-Based Activated Carbons

The EUO wood was collected from the experimental field of Northwest A&F University, Yangling, Shaanxi, China. The bark and leaves of the wood were removed, and the crushed wood was sifted for particles of 40–60 mesh. The screened EUO wood particles (10.00 g) were mixed up together with deionized water (80 mL) in a Teflon lined high-pressure reaction kettle. Then, it was held in a muffle furnace for 1 h at 170 °C. After the hydrothermal reaction, the obtained tan solid was impregnated equably with H_3_PO_4_ solution (10%, 15%, 25%, 35%, 40%, 45%, 50%, and 55%); the mass ratios of EUO wood to H_3_PO_4_ was 1:2, 1:3, 1:4, and 1:5, respectively. After drying at 105 °C, the EUO meal was placed in a tubular furnace to prepare AC in a nitrogen atmosphere. For all the impregnated feedstock, the constant temperature growth rate of heating was set at 5 °C·min^−1^; after reaching the target temperature (350 °C, 400 °C, 450 °C, and 500 °C), the corresponding activation retention time (2 h, 4 h, 6 h, and 8 h) was maintained. The products were evenly mixed with 1% hydrochloric acid at a temperature of 105 °C for 50 min, and then washed until the filtrate was neutral. The ACs were prepared by single factor experiment with 25% (the concentration of H_3_PO_4_), 1:4 (the mass ratios of EUO wood to phosphoric acid), 2 h (activation temperature), and 400 °C (activation retention time) as the control group, which were labeled as EC-X% (X% was equivalent to the mass fraction of phosphoric acid, EC was the Eucommia-based wood-activated carbon), EC-1:X (1:X was the solid–liquid ratio), EC-X h (X h was the activation maintaining time), and EC-X (X was the activation temperature), respectively. The thermogravimetric analysis was applied for exploring the thermal stability and composition of the feedstock; the nitrogen flow rate is 20 mL·min^−1^, and the purity was 99.9%. The pore size distribution and specific surface area of the activated carbon were analyzed by the specific surface area analyzer (V-SORB 2800P), the sample was tested at −196 °C with nitrogen (99.9%) and helium (99.9%) as auxiliary gases. The crystal structure of the sample was examined by X-ray diffraction (XRD, Bruker D8 ADVANCE A25) with a Cu Kα radiation (λ = 1.5418) in a 2θ range from 10° to 80°. The Raman spectrum analysis of the samples was carried out by scanning the Raman spectrometer (Horiba Jobin Yvon, Longjumeau, France) at ambient temperature on a Raman microscope, and analyzed on the data processing software of the instrument configuration. The surface functional groups of the activated carbon were investigated by X-ray photoelectron spectroscopy (XPS) with Al Kα radiation, and the pore structure imaging was performed by the transmission electron microscope (TEM) [26].

### 2.2. Preparation and Electrochemical Test of Electrode Plates of Supercapacitor

The ECs, carbon black, and polytetrafluoroethylene (PTFE) were mixed with a quality ratio of 85:10:5. After that, 9.5 mg mixture was evenly applied to the electrode collector (stainless steel mesh) and pressed with the pressure of 1 Mpa. The carbon-based electrode was set as the working electrode, the platinum foil as the counter electrode and the Hg/Hg_2_SO_4_ electrode as the reference electrode. The obtained working electrode was electrochemically measured in a three-electrode system. The cyclic voltammetry (CV), galvanostatic charge-discharge (GCD), and electrochemical impedance spectroscopy (EIS) were measured in 1 mol·L^−1^ H_2_SO_4_ solution at an electrochemical workstation (CHI660E, Shanghai, China). The potential windows tested for CV range from −0.8 V to 0.4 V, the charge and discharge current density for CGD was 0.2 to 2 A·g^−1^ and the EIS measurement frequency range from 100 kHz to 10 MHz, the alternating current signal amplitude at open potential was 5 mV.

The specific capacity *Cs* (specific capacitance) of the electrode was calculated by using the CV curve, and analyzed by equation,

*Cs* = *Q*/(*m* × Δ*v*) (1)

and the equation of *Cs* calculation by GCD was as follows,

*Cs* = *I* × Δ*t*/(*m* × Δ*v*) (2)

where *Q* was the quantity of electric charge, ∆*v* was the change of the potential window in the discharge, Δ*t* was the charge and discharge time, and m was the mass of the whole electrode (g) [27,28].

### 2.3. Characterization and Methods

In this study, ACs were prepared from EUO-wood by H_3_PO_4_ activation, the effect of H_3_PO_4_ concentration, activation time, temperature, and solid–liquid ratio on the amorphous properties of ACs were systematically investigated. The surface and internal structure of ACs were observed by TEM and characterized by Raman spectrum, XRD, specific surface area analyzer, and XPS. The pore size distribution and the specific surface area of ACs were determined by the Brunauer–Emmett–Teller (BET) and Barrett–Joyner–Halenda (BJH) method (Tristar II 3020, Micromeritics Instrument Corporation, Norcross, GA, USA). The electrochemical properties of ACs were analyzed by CV, EIS, and GCD, respectively. Moreover, the correlation between pore structure and the specific surface area of ACs and their electrochemical properties as supercapacitor electrodes was also further demonstrated.

### 2.4. Economic Assessment

The operating costs, based on the unit of specific capacity, were analyzed in an economic assessment of the low-temperature H_3_PO_4_ activation process of the Eucommia wood-based carbon for supercapacitor preparation under different process conditions. This cost involves expenditures for electricity consumption and material use; however, it does not include equipment cost, labor cost, and overhead production cost. The electricity consumption and material use of the economic evaluation are listed in Table 1.

## 3. Result and Discussion

### 3.1. Structure and Morphology Characterization of ECs

TGA of the EUO-wood was shown in Figure S1a, which exhibited three stages during the process of pyrolysis. The dehydration stage of EUO-wood (0–150 °C) with 10.92% weight loss, which demonstrated the evaporation of water, occurred in the EUO-wood. In the range of 150–350 °C, a sharp weight loss stage can be observed, which corresponded to the pyrogenic decomposition of hemicellulose. In the third thermal decomposition stage (360–700 °C), the curve flattened out gradually, which was attributed to the stable pyrolysis of lignin carbon. No peak appeared in the subsequent pyrolysis process (curve of DTG) on account of the slow decomposition of lignin. In Figure S1b, the initial pyrolysis temperature (102 °C) of the EUO-wood infiltrated by H_3_PO_4_ was lower than that in the feedstock (150 °C). Because of the catalytic dehydration of phosphoric acid, the hemicellulose and cellulose occurred cross-linked with H_3_PO_4_ at low temperature, which started at 102 °C and ended at 308 °C. Before the heat treatment temperature reached 200 °C, significant aromatization occurs in wood impregnated with phosphoric acid [29]. In the temperatures’ scope from 308 °C to 700 °C, sp2 carbon atoms in cellulose and hemicellulose were further atomized, and lignin was catalyzed by H_3_PO_4_ to promote the formation of the graphite-like basic microcrystalline [30]. Therefore, the coating and dehydration of H_3_PO_4_ can promote the decomposition of the EUO-wood at a low temperature, which inhibited the formation of volatile compounds of carbon, and improved the yield of ACs (Table 2).

**Figure 1:**
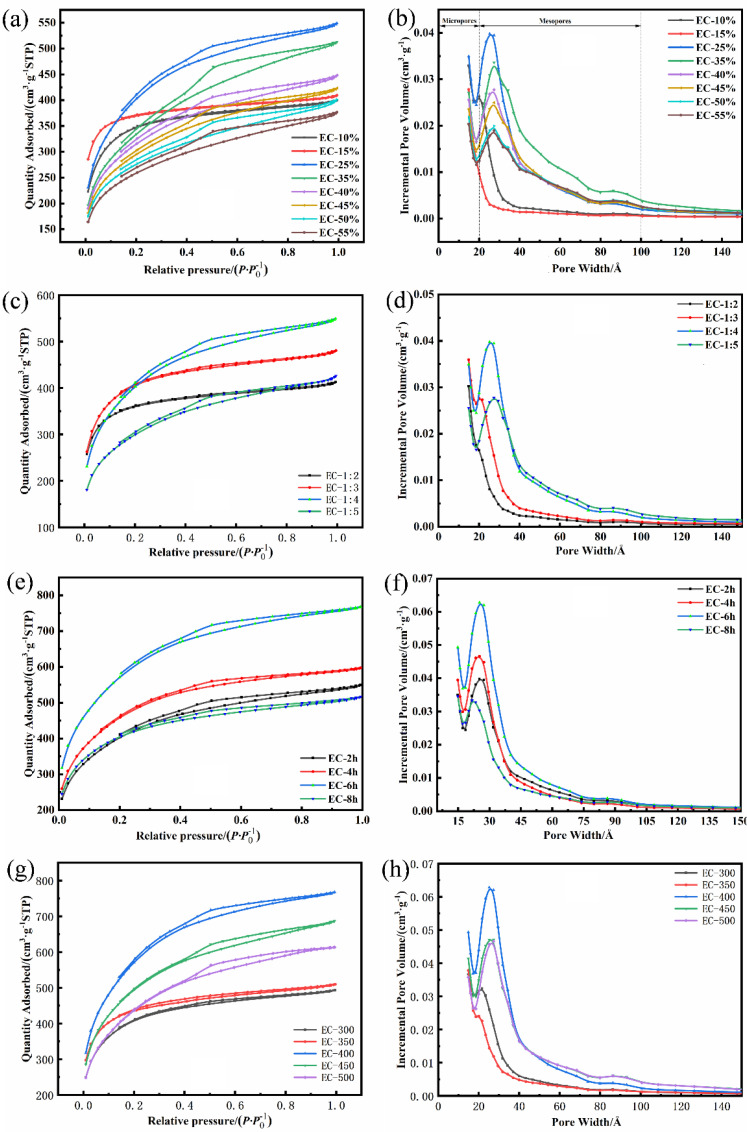
(**a**,**c**,**e**,**g**) Nitrogen adsorption-desorption curves of ECs; (**b**,**d**,**f**,**h**) Pore size distribution curves of ECs.

The pore structure was evaluated by the N_2_ adsorption-desorption, which was revealed in Figure 1 and Table 2. According to Figure 1a,c,e,f, the major adsorption occurred in the relative menubar (0–0.2), and the subsequent adsorption capacity tended to plateau in the middle and high relative pressure region, which demonstrated the existence of micropores in the ECs. In addition, based on the classification of IUPAC, the isothermal adsorption curves of ACs (EC-10%, EC-15%, EC-1:2) belonged to type I isotherm without an obvious hysteresis loop [31], which contained the larger micropore volume than other samples according to the corresponding pore distribution diagram (Figure 1b,d). By the hysteresis loops of other samples, the ECs can be observed to possess the mesoporous structure; therefore, the N_2_ adsorption-desorption curves belonged to the type IV isotherms [32]. The proportion of the mesoporous volume could affect the specific surface area of AC directly. As the mesoporous proportion was 96.4%, the maximum specific surface area reached 2033.87 m^2^·g^−1^. In Figure 1a, with the increase in the H_3_PO_4_ concentration, the majority of H_3_PO_4_ molecules cross-linked with the hydroxyl groups in the EUO-wood to form the heat-resistant phosphate ester linkage. At low current densities, the cross-linking of phosphoric acid with biopolymers through phosphate ester bonds prevents the contraction of the cell wall during heat treatment, which contributes to developing a pore structure of the phosphoric acid-activated carbon [33,34]. Therefore, the well-developed structure (the mesoporous ratio was 93.9%) was formed on account of a moderate concentration of H_3_PO_4_ (25%). When the concentration of H_3_PO_4_ exceeded 25% to 55%, the specific surface area and the mesoporous ratio reduced to 888.95 m^2^ and 71.7%, respectively. It can be speculated that excessive crosslinking products generated uneven heating, and the shrinkage and expansion can synthesize macropore, which reduced the mesoporous ratio and specific surface area. Figure 1c,d demonstrated the influence of the mass ratios of EUO-wood to H_3_PO_4_ on the N_2_ adsorption and pore diameter distribution of ECs. According to the variations of the pore structure and specific surface area (Table 2), it can be inferred that the dose of H_3_PO_4_ came into a similar effect on the concentration of H_3_PO_4_ and on its internal structure.

As the activation time prolonged from 4 h to 6 h (Figure 1e,f), the hysteresis loop was expanding gradually with the increased adsorption capacity. It was benefited by the micropore expansion into mesoporous, which accounted for the effective increase in the specific surface area and mesoporous volume (1613.28 m^2^·g^−1^ to 2300.87 m^2^·g^−1^, 0.70 cm^3^·g^−1^ to 1.07 cm^3^·g^−1^) with the increased pore-produced rate on account of the extension of activation time. As the activation time increased to 8 h, more aromatic carbon atoms were involved in the activation reaction, and the carbon skeleton was etched when the finite number of carbon atoms in the active site was over-consumed. The rate of pore-producing was slower than that of pore-enlarging, which led to the specific surface area being reduced to 1048.17 m^2^·g^−1^ with the collapse of the pore wall; meanwhile, the yield of AC was decreased from 93% (EC-6 h) to 87.3% (EC-8 h). In Figure 1g,h, the relationship between activation temperature and the change of pore structure can be verified. During the activation process, most carbon atoms at the active site were lacking enough activation energy at 350 °C to reach the activation condition, which could react with H_3_PO_4_ to form pores. The total pore volume was 0.54 cm^3^·g^−1^, and the specific surface area was 1446.05 m^2^·g^−1^. It can be noticed that when the temperature went up to 400 °C, the rate of yield decreased drastically (from 52.7% of EC-350 to 43.8% of EC-400). During this temperature range, the activation reaction was intense and magnified the carbon consumption, which contributed to the number of carbon atoms that entered the activation state gradually. As the activation temperature increased to 500 °C, the internal structure of AC collapsed with the carbon skeleton continuing to be ablated. Moreover, the formed phosphate ester bond (condensation of H_3_PO_4_ and a hydroxyl group) was fractured at a relatively high temperature, leading to the increase in average pore diameter (2.86 nm) and the decrease in specific surface area (1553.15 m^2^·g^−1^). According to Table 2, the obtained ECs can be defined as a hierarchical-structure mesoporous carbon, which can facilitate the transport and storage of ions, i.e., supercapacitors’ applications.

The visual pore structure of ECs was observed via the TEM method, as shown in Figure S2. The typical sample possessed a cellular structure and exhibited an obvious layer structure. The pores between layers were connected mutually, forming a three-dimensional-developed layers pore structure with a maximum specific surface area (2033.87 m^2^·g^−1^). On the contrary, another representative sample (EC-10%) demonstrated a large number of layer-like pores, which was smaller than that in EC-400. Therefore, the main difference between EC-400 and EC-10% was that the former (EC-400) obtained the amorphous structure with obvious internal defects, which was expected to be a high-performance electrode material.

**Figure 2:**
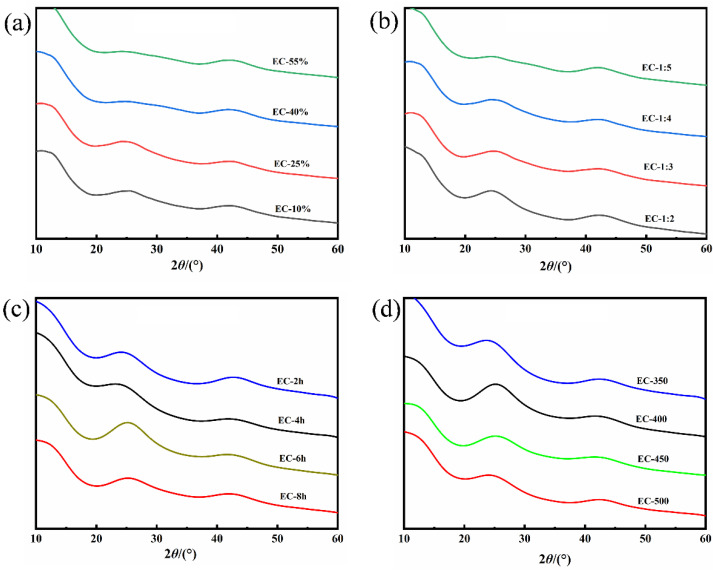
(**a**,**b**,**c**,**d**)The XRD spectra of the ECs.

**Figure 3:**
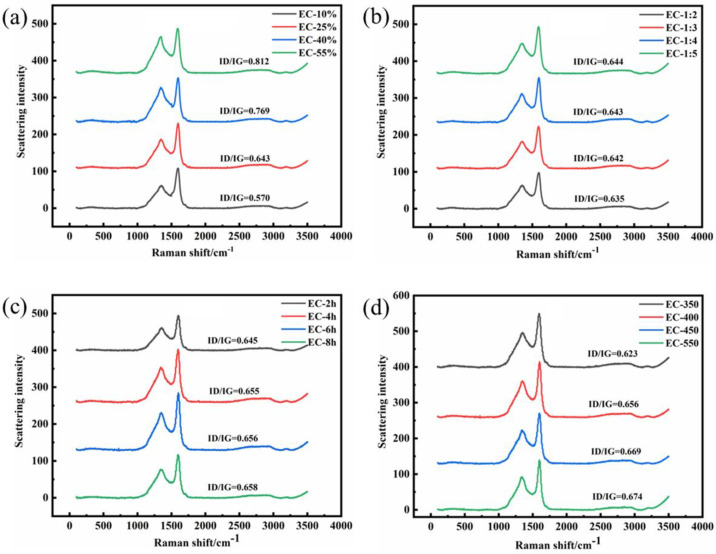
(**a**,**b**,**c**,**d**)The Raman spectra of the ECs.

AC was considered to be composed of a regular lamellar crystal structure and irregular carbon. XRD technique is an effective means to explore the microcrystalline structure of ACs. Figure 2 was the XRD pattern of ECs. It can be observed that there are two absorption peaks at 24° and 43°, corresponding to the (002) and (100) crystal surfaces, respectively [1]. The etching effect of H_3_PO_4_ will destroy the relatively regular wafer crystal structure in the EUO wood, causing the crystal plane diffraction peak to gradually flatten, and forming amorphous AC with flourishing pores and prodigious specific surface area. The increase in H_3_PO_4_ concentration and dosage, activation temperature, and activation time weakened the diffraction peak of the 002 crystal surface gradually. These phenomena were consistent with the analysis of the adsorption and desorption and pore distribution curves in the aforementioned part (Figure 1). On the whole, the peaks of XRD patterns were broad generally, which implied the low graphitization and irregularity of ECs with an abundant amorphous structure. Moreover, Raman spectroscopy was also employed to analyze the amorphous structure of the ECs. The Raman spectrums of all samples demonstrated typical absorption peaks at 1350 cm^−1^ and 1600 cm^−1^, corresponding to the D band and G band of carbon singly. The appearance of the D band was attributed to the disordered characteristics caused by lattice defects and impurities of carbon atoms, while the G band represented the stretching vibration in the hybrid plane of carbon atoms, sp2 [35]. Normally, the graphitization degree of carbon material was measured by the strength ratio of the D band to the G band (I_D_/I_G_, where I_D_ and I_G_ are the D-band and G-band Raman intensities) [36,37]. As shown in Figure 3a,b, when the pyrolysis time and temperature were fixed (400 °C for 2 h), the graphitization degree was affected by the concentration and dosage of phosphoric acid. It can be concluded that the presence of increased H_3_PO_4_ solution can promote the formation and expansion of pores during the activation process, which revealed the amorphous of ACs. On account of the reduction in graphitization (the ratio of I_D_/I_G_ in Figure 3a,b), the defects of AC were magnified with the increased average pore size. The effect of heat preservation time and activated temperature on the crystallinity of AC in the pyrolysis process was demonstrated in Figure 3c,d. The ratio of I_D_/I_G_ was 0.645 (EC-2 h), 0.655 (EC-4 h), 0.656 (EC-6 h) and 0.658 (EC-8 h), respectively, in Figure 3c. Meanwhile, the ratio of I_D_/I_G_ severally was 0.648, 0.656, 0.669, and 0.674 for ECs with the activation temperature as a variable (Figure 3d). It can be observed that the ratio of I_D_/I_G_ was magnified slightly with the extension of the activation time and the increase in activation temperature, lessening the graphitization of AC to some extent. Peculiarly, significant otherness can be obtained by analyzing the strength of the D-band. The D-band represents the defect site on the structure of the AC based on the Raman spectrums. The maximum strength of the D-band (101.7) and G-band (155) can be noticed, as the H_3_PO_4_ concentration was 25%, the mass ratio feedstock-activator was 1:4, the activation time was 6 h, and the activation temperature was 400 °C, which was attributed to the defects in the carbon atom crystal, and a highly porous disordered structure was formed. Therefore, the amorphous structure of AC was expected to possess the capacity to store a large amount of charge.

**Figure 4:**
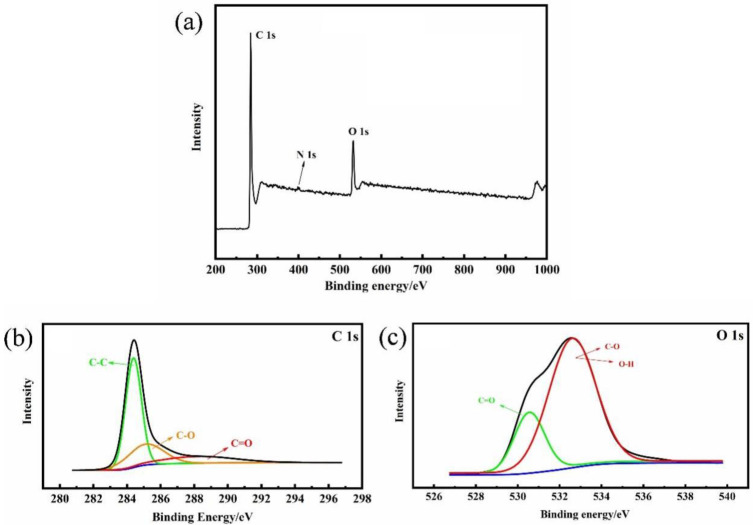
(**a**) XPS pattern of EC-400; (**b**) high resolution C 1s; and (**c**) O 1s.

The surface chemical information of AC prepared by this process of conditions was further analyzed (Figure 4). In Figure 4a, there were three peaks represented: C 1s, N 1s and O 1s, which were located at 284.5 eV, 404.8 eV, and 532.8 eV, respectively. Among them, the proportion of nitrogen atoms was extremely low, and the subsequent high-resolution atlas of the N 1s was not analyzed. Three high-resolution peaks were observed in the deconvolution of C 1s. The 284.6 eV was corresponding to the sp2 carbon and C-C bond, the other two peaks were assigned to the C-O bond and C=O bond (285.2 eV and 288.7 eV). For O 1s’ high-resolution spectra, two distinct peaks can be observed, which were related to the C=O bond (530.6 eV) and C-O, O-H bond (532.7 eV), severally [38]. Oxygenated functional groups are generally present on the surface of the material, which has demonstrated significant effects on the electrochemical properties of the material. When using aqueous electrolytes, the polarization of these surface functional groups could improve the surface wettability of carbon materials, which may reduce the impedance in the application of supercapacitors and enhance the electrochemical performance of the electrode materials [39].

### 3.2. Electrochemical Characterization of ECs

**Figure 5:**
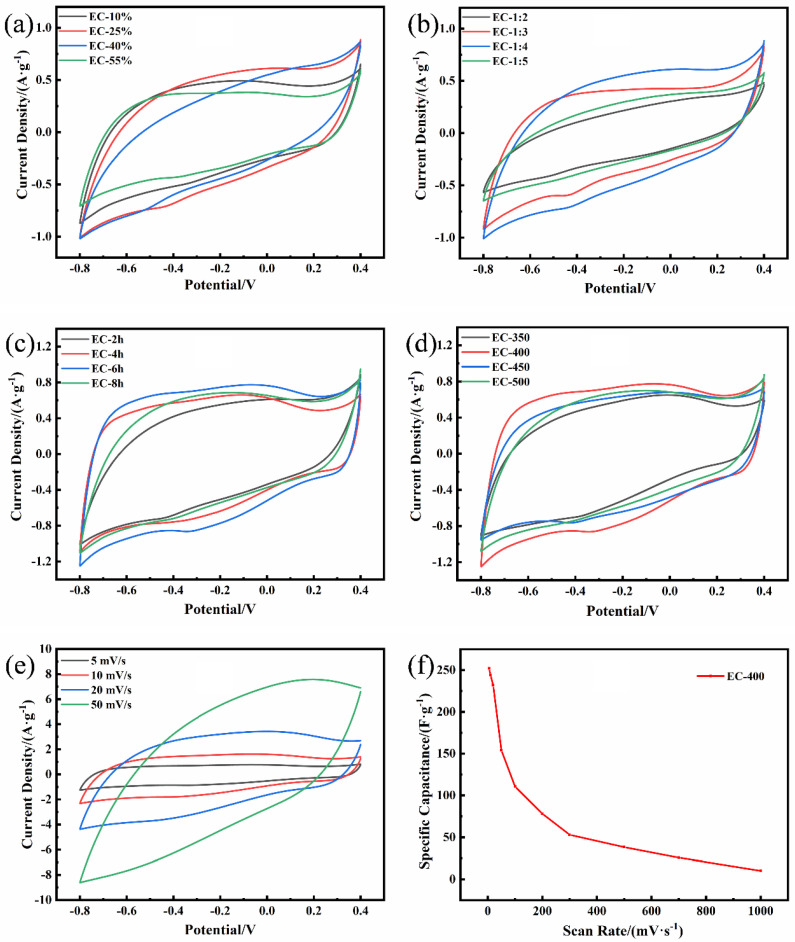
(**a**–**d**) The CV curves of ECs at 5 mV·s^−1^; (**e**) the CV curves of EC-400 at different scan rates; (**f**) the specific capacitance of EC-400 at different scan rates.

The electrochemical performance of the ACs-based electrodes was tested in a three-electrode system with 1M H_2_SO_4_ electrolyte. The cyclic voltammetry curve revealed the relationship between the response current and voltage, which can reflect the electrochemical behavior of the electrode surface to evaluate the electrochemical behavior. The shape of all the CV curves was approximately quadrilateral, indicating that the main capacitance of the supercapacitor was provided by the double electric layer. Some puny redox peaks can be found in the curves, which were generated through the redox reaction of the electrolyte with the surface groups and provided a portion of the pseudocapacitance (the appearance of surface functional groups was verified by XPS) [40]. In Figure 5, the curves of EC-10%, EC-1:2, and EC-1:3 exhibited more obvious deformation (deviated from the quasi-rectangular), and specific capacitance (140 F·g^−1^, 75 F·g^−1^, and 140 F·g^−1^) was smaller than those of others. These samples had numerous micropores in common, leading to the increase in the diffusion resistance of electrolyte ions in the pores and the decrease in electrochemical performance, which can be verified by the subsequent EIS analysis. The ECs-based electrode materials synthesized by different activation processes (Figure 5e–d) displayed different specific capacitances (Table 3), which were associated with the region enclosed by the CV curve, relating with the pore structure and specific surface area of AC. The reasonable pore distribution generated a large specific surface area and enhanced the charge-storage ability, increasing the specific capacitance. Through the comparison and analysis, EC-400 showed the maximum specific capacitance (252 F·g^−1^) when the scanning rate was 5 mV·s^−1^; it was selected for CV analysis at different scanning rates (Figure 5e). The adsorption and desorption of electrolyte ions in AC micropores cannot be operated completely in the process of high-speed scanning, and the specific capacitance provided by the micropore presented a trend of a gradual decrease with the rise of scanning rate (5 mV to 50 mV). Moreover, the increasing scanning rate resulted in the number of surface functional groups reacting with the electrolyte decreasing sharply and weakening the redox intensity of the peak; therefore, the capacitance was mainly provided by the double-layer. Meanwhile, the total specific capacitance decreased from 252 F·g^−1^ to 154 F·g^−1^ when the scanning rate increased from 5 to 50 mV·s^−1^; all the CV curves of ACs were exhibited in Figures S3 and S4 and the parameters were exhibited in Table 3. As the scanning rate continued to increase, the specific capacitance reduction rate gradually flattened out (Figure 5f); it was indicated well that the stability of mesopores in the electronic transfer was better than that of the micropores. At low current densities, the micropores can achieve high charge storage capacity. At high current densities, electrolyte ions can still be transported rapidly in the mesopores. A well-distributed pore size structure makes an important contribution to the rate performance of activated carbon electrodes [41]. Meanwhile, compared to activated carbons prepared from other biomass (Soya Beans, Binata, Tree bark, Brussel, Rotten carrot, Cottonseed meal, Tamarindus indica Fruit Shell, Baobab shell, and Olive residue), the EC-400 exhibited a high specific capacitance (Table S2).

**Figure 6:**
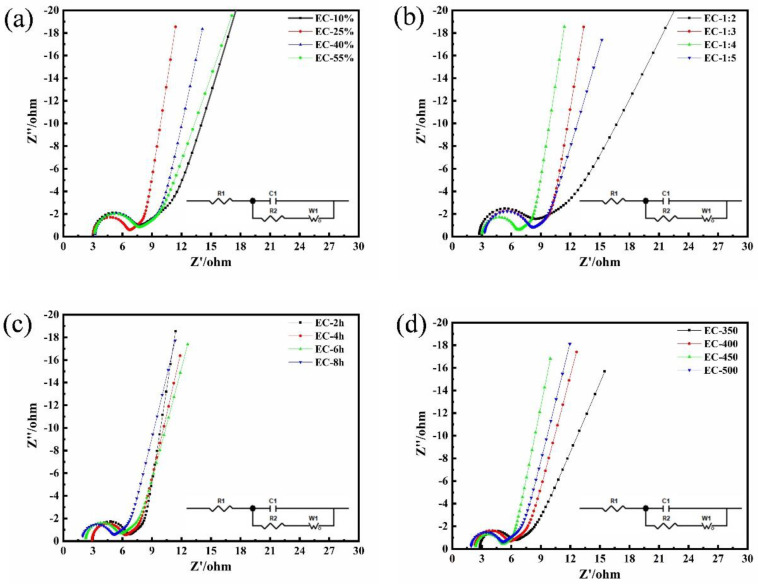
(**a**–**d**) The EIS curves of ECs.

The sinusoidal potential wave generated the response current of the three-electrode system, and the EIS was plotted by measuring the response function of the electrode system in the wide frequency range (0.01 Hz to 100,000 Hz) (Figure 6). The EIS can not only display the series resistance of the AC-based electrode but also analyzes the dynamics of the charging and discharging process. In general, the EIS consists of the real part Z’ and the imaginary part Z”, and divides into three blocks (high-frequency region, intermediate frequency region (impedance region), and low-frequency region). The inset equivalent circuit was shown in Figure 6c that included the equivalent circuit parts of *R_s_* (the intrinsic resistance of the substrate or electrolyte resistance), *R_ct_* (the charge transfer resistance), and W (the Warburg impedance). The spectrum of the high-frequency region exhibited a semicircle shape, which matched the *R_ct_*. The ECs all exhibit smaller *R_s_* and *R_ct_*, as shown in Table 4. It can be found that the average pore size of the sample was inversely proportional to the magnitude of the *R_ct_*. In detail, the smallest *R_ct_* was 2.488 Ω and the largest *R_ct_* was about 4.379 Ω. The electron conductivity was limited by the magnitude of *R_ct_*, which indicated that the excellent conductivity of the EUO-based electrode materials was conducive to electrochemical performance. In the intermediate frequency region, the impedance curve presented a line of about 45°, which implied the ion-diffused resistor (R_w_) in the pores of the carbon electrode [42]. It also revealed a transition from resistive behavior to capacitive behavior. The low-frequency region was formed due to the charge-discharge performance of the porous structure surface; the value of the frequency was inversely proportional to the magnitude of the slope, expressing the typical capacitance performance in the electrodes. For an ideal electrode material, the curve was a straight line perpendicular to the *X*-axis. The ECs exhibited typically capacitive characteristics on account of the well-developed mesoporous structure with a large specific surface area except for EC-10%, EC-55%, EC-1:2, and EC-350. These results suggested that numerous mesoporous and well-developed pore structures could create a satisfactory contact between electrode and electrolyte.

**Figure 7:**
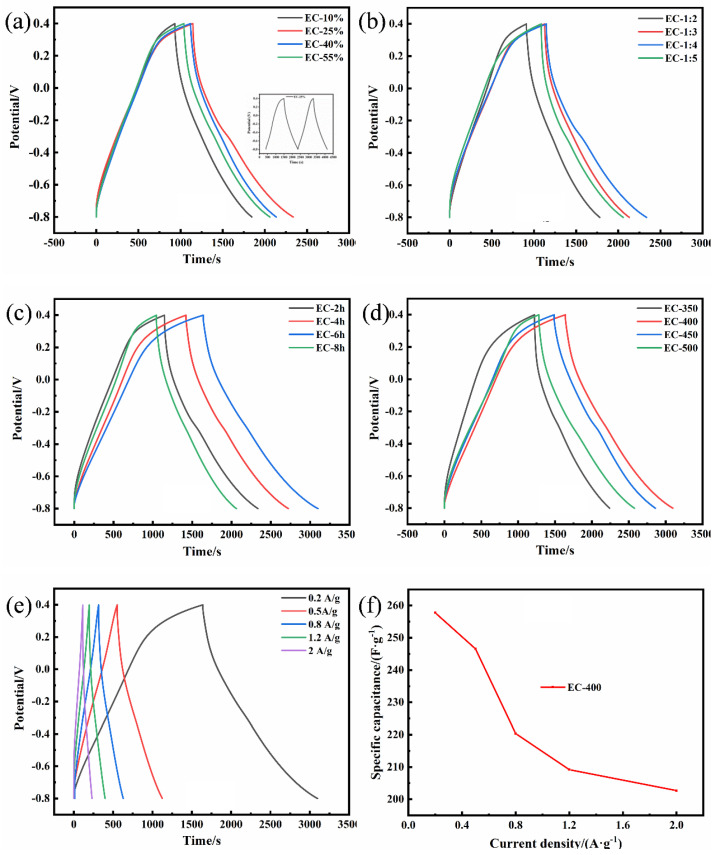
(**a**–**d**) GCD curve of ECs at 0.2 A·g^−1^; (**e**) GCD curve of ECs at different current densities; (**f**) the specific capacitance of EC-400 at different current densities.

The GCD profiles of ECs were demonstrated in Figure 7 (at 0.2 A·g^−1^). According to the curve extension of EC-25% in Figure 7a, after the first charge-discharge, the curve revealed a regular shape of an isosceles triangle, demonstrating excellent charge and discharge efficiency. The curve of the first charge had a pronounced curvature, which contributed to the carbon surface, which contained a certain number of functional groups. The H_2_SO_4_ electrolyte will undergo an irreversible chemical reaction with some active functional groups, consuming a certain amount of charge and prolonging the charge time. After the electrolyte fully wetted the AC-based electrode, the capacitance tended to be stable. To eliminate the instability of the initial charge, the capacitance of the AC-based electrode was calculated by the discharge process. The period of discharge curve can reflect the capacitance (Table S1), which was proportional to it. This specific capacitance was consistent with the change of pore volume and specific surface area and represented a correlation among them. When the H_3_PO_4_ concentration was relatively low, the pore structure of AC was not developed enough, resulting in a lower specific surface area. Therefore, the ACs (EC-10%, EC-1:2) cannot store the ions effectively in the electrolyte, accounting for the charge quantity released during the discharge that tailed off and lessened the capacitance naturally [43]. While the mass ratio of solid–liquid was 1:4 with 25% phosphoric acid, the obvious amorphous structure was beneficial to the electrolyte ion adsorption and the charge desorption, promoting the specific capacitance to rise to 194.15 F·g^−1^. With the increase in the H_3_PO_4_ solution, the appearance of macropores led to a decrease in pore volume, surface area, and specific capacitance. The activation time and temperature were taken as independent variables (Figure 7c,d), the charge-discharge period of the working electrode reached 3098 s (257.8 F·g^−1^) when the activation temperature was 400 °C and maintained 6 h, and the highest specific surface area (2033.87 m^2^·g^−1^) appeared, which confirmed that the large specific surface area was conducive to the charge storage and release. In the meantime, the emergence of the IR (internal resistance) drop can be noticed. The IR was caused by the unstable material properties at the beginning of the discharge; on account of storing some virtual charges, a voltage drop occurs at the discharge moment [44]. This phenomenon was attributed to the binding between the active substances in the electrode system and the mechanical and electrochemical properties of the electrode materials. The IR drops of all the ECs were slight (0.020–0.030 V), which implied that the structure of the AC facilitated the storage of the charge and enhanced the stability of the material. Figure 7e expressed the different charge and discharge periods of EC-400 at the current density of 0.2 A·g^−1^, 0.5 A·g^−1^, 0.8 A·g^−1^, and 1.2 A·g^−1^; the charge and discharge time was shortened with the enhancement of the current density. This phenomenon can be explained by the fact that the diffusion of electrolyte ions to the microporous structure at a high charging rate is restricted when the current density increases. This will lead to a decrease in capacitance (Table S1) [45]. On the contrary, the electrolyte ions are more likely to enter the mesoporous structure at a low charging and discharging rate. With the increase in current density, the IR drop also increased from 0.045 V to 0.214 V. It demonstrated that the discharge capacitance (DC) decreased with the rise of the current density (Figure 7f). These results were identical to CV curves, which may be due to the developed mesopores’ structure of the active materials, resulting in the high accessibility of electrolyte ions to the electrode surface with high capacitance.

To probe the service life of ECs, the EC-10% and EC-400 were selected to be the candidates for long-term cycling studies (Figure S5). The discharge capacitance of both revealed a decreasing trend at an initial stage and then it leveled off. The loss ratio of capacitance was 10.2% and 8.6%, respectively, after 10,000 cycles, which suggested that the EUO-AC-based electrode materials possessed manifest satisfactory long-term stability. Compared to the EC-10%, the EC-400 obtained better durability, which may be attributed to the stable mesoporous structure than that of others. Therefore, the above experiments confirmed that the EC-400 electrode was the advisable material to apply in supercapacitors. The EUO wood was directionally converted to mesoporous ACs via low-temperature chemical treatment, which made the EUO wood for enforceable application in supercapacitors.

### 3.3. Economic Assessment

For commercial application, a technical-economic evaluation was promoted for the use of H_3_PO_4_ activation from an industrial perspective. Table 5 listed the operating costs of the low-temperature H_3_PO_4_ activation process of Eucommia wood-based carbon for supercapacitor preparation, which were under different process conditions based on units of specific capacity. The supercapacitor preparation using the EC-400 condition had the best economic feasibility with respect to electricity storage. The electricity for drying had the highest contribution to the cost (54%), electricity for activation is responsible for 26% of the cost, and electricity for a hydrothermal reaction for the cost is made up of 13%. The economic feasibility of the supercapacitor preparation using the EC-450 condition and the EC-500 condition was fairly good. The electricity for drying was the major single contributor (54%). Other important contributors were electricity for activation (26%) and electricity for hydrothermal reaction (13%). However, the supercapacitor preparation using the EC-1:2 condition demonstrated the worst economic feasibility. The cost was dominated by electricity for drying (57%). Other relevant contributors were electricity for activation (26%) and electricity for hydrothermal reaction (13%). Therefore, EC-400 was the most recommended process condition for the supercapacitor preparation via the low-temperature H_3_PO_4_ activation. Physical activation is less corrosive than chemical activation but needs a much longer time. In the one-step pyrolysis method, the activation process consolidates the carbon framework. Subsequently, the chemical activation process saves more pyrolysis time.

The use of chemicals facilitated a degree of activation effectiveness because of the nonactive surface of the charcoal, while also resulting in a large cost of electric energy consumption (93%). The reduction in electric energy consumption will help decrease the cost. For example, the electricity for the drying and hydrothermal reaction can be replaced with natural gas combustion for the drying and hydrothermal reaction. Moreover, it is surprising that EC-1:2 preparation consumed as much electricity as EC-400, EC-450, and EC-500 preparation. However, EC-1:2 preparation consumed less H_3_PO_4_ than EC-400, EC-450, and EC-500 preparation, and H_3_PO_4_ for the costs only makes up less than 0.0001%. This probably shows that H_3_PO_4_ is an extremely important factor for the electrode materials preparation. Maybe a moderate increase in H_3_PO_4_ use will contribute to a remarkable improvement in supercapacitor performance but will not lead to a remarkable rise in operating costs. Moreover, a high product yield for the chemical activation method results in a low preparation cost and the obtained activated carbon with a high SSA is expected to have a high selling price.

## 4. Conclusions

H_3_PO_4_ activation at a low-temperature was an effective way to prepare ECs; the liquid–solid ratio, activation time, and temperature were the key factors to determine the physico-chemical property of the ECs. The satisfactory mesoporous EC exhibited a well-developed mesoporous structure (1.07 cm^3^·g^−1^), with the highest specific surface area (2033.87 m^2^·g^−1^) among all ECs under different process conditions. The presence of mesoporous facilitated ion transport enhanced the electrochemical performance. Moreover, on account of the high specific surface area, the excessive contact between the electrode and electrolyte provided a larger specific capacitance, which was up to 252 F·g^−1^ and had long-term stability (capacitance retention of 91.4% after 10,000 cycles). Moreover, the EC-400 possessed the best economic feasibility among all the ECs. Thus, EUO wood is a value-added feedstock that can be converted into ACs-based electrode materials for supercapacitors’ application.

## Figures and Tables

**Table 1:** The electricity consumption and material used for different process conditions.

	EC-10%	EC-25%	EC-40%	EC-55%	EC-1:2	EC-1:3	EC-1:4	EC-1:5	EC-2h	EC-4h	EC-6h	EC-8h	EC-350	EC-400	EC-450	EC-500
EUO (ton)	1.00 × 10^−5^	1.00 × 10^−5^	1.00 × 10^−5^	1.00 × 10^−5^	1.00 × 10^−5^	1.00 × 10^−5^	1.00 × 10^−5^	1.00 × 10^−5^	1.00 × 10^−5^	1.00 × 10^−5^	1.00 × 10^−5^	1.00 × 10^−5^	1.00 × 10^−5^	1.00 × 10^−5^	1.00 × 10^−5^	1.00 × 10^−5^
Deionized water (L)	8.00 × 10^−2^	8.00 × 10^−2^	8.00 × 10^−2^	8.00 × 10^−2^	8.00 × 10^−2^	8.00 × 10^−2^	8.00 × 10^−2^	8.00 × 10^−2^	8.00 × 10^−2^	8.00 × 10^−2^	8.00 × 10^−2^	8.00 × 10^−2^	8.00 × 10^−2^	8.00 × 10^−2^	8.00 × 10^−2^	8.00 × 10^−2^
Electricity for hydrothermal reaction (kwh)	4.00	4.00	4.00	4.00	4.00	4.00	4.00	4.00	4.00	4.00	4.00	4.00	4.00	4.00	4.00	4.00
H_3_PO_4_ (kg)	1.60 × 10^−5^	4.00 × 10^−5^	6.40 × 10^−5^	8.80 × 10^−5^	2.00 × 10^−5^	3.00 × 10^−5^	4.00 × 10^−5^	5.00 × 10^−5^	4.00 × 10^−5^	4.00 × 10^−5^	4.00 × 10^−5^	4.00 × 10^−5^	4.00 × 10^−5^	4.00 × 10^−5^	4.00 × 10^−5^	4.00 × 10^−5^
Electricity for drying (kwh)	1.67 × 10^1^	1.67 × 10^1^	1.67 × 10^1^	1.67 × 10^1^	1.67 × 10^1^	1.67 × 10^1^	1.67 × 10^1^	1.67 × 10^1^	1.67 × 10^1^	1.67 × 10^1^	1.67 × 10^1^	1.67 × 10^1^	1.67 × 10^1^	1.67 × 10^1^	1.67 × 10^1^	1.67 × 10^1^
Electricity for activation (kwh)	8.00	8.00	8.00	8.00	8.00	8.00	8.00	8.00	8.00	1.60 × 10^1^	2.40 × 10^1^	3.20 × 10^1^	8.00	8.00	8.00	8.00
Hydrochloric acid (L)	2.00 × 10^−1^	2.00 × 10^−1^	2.00 × 10^−1^	2.00 × 10^−1^	2.00 × 10^−1^	2.00× 10^−1^	2.00 × 10^−1^	2.00 × 10^−1^	2.00 × 10^−1^	2.00 × 10^−1^	2.00 × 10^−1^	2.00 × 10^−1^	2.00 × 10^−1^	2.00 × 10^−1^	2.00 × 10^−1^	2.00 × 10^−1^
Electricity for heating (kwh)	9.17 × 10^−1^	9.17 × 10^−1^	9.17 × 10^−1^	9.17 × 10^−1^	9.17 × 10^−1^	9.17 × 10^−1^	9.17 × 10^−1^	9.17 × 10^−1^	9.17 × 10^−1^	9.17 × 10^−1^	9.17 × 10^−1^	9.17 × 10^−1^	9.17 × 10^−1^	9.17 × 10^−1^	9.17 × 10^−1^	9.17 × 10^−1^
Wash Water (L)	4.00	4.00	4.00	4.00	4.00	4.00	4.00	4.00	4.00	4.00	4.00	4.00	4.00	4.00	4.00	4.00
Carbon black (kg)	1.31 × 10^−4^	1.31 × 10^−4^	1.31 × 10^−4^	1.31 × 10^−4^	1.31 × 10^−4^	1.31 × 10^−4^	1.31 × 10^−4^	1.31 × 10^−4^	1.31 × 10^−4^	1.31 × 10^−4^	1.31 × 10^−4^	1.31 × 10^−4^	1.31 × 10^−4^	1.31 × 10^−4^	1.31 × 10^−4^	1.31 × 10^−4^
PTFE (kg)	6.54 × 10^−5^	6.54 × 10^−5^	6.54 × 10^−5^	6.54 × 10^−5^	6.54 × 10^−5^	6.54 × 10^−5^	6.54 × 10^−5^	6.54 × 10^−5^	6.54 × 10^−5^	6.54 × 10^−5^	6.54 × 10^−5^	6.54 × 10^−5^	6.54 × 10^−5^	6.54 × 10^−5^	6.54 × 10^−5^	6.54 × 10^−5^

**Table 2:** Pore size distribution and yield of activated carbon in the different activation processes.

Sample	BET ^a^ /(m^2^·g^−1^)	D_average_^b^ /Å	V_total_^c^ /(cm^3^·g^−1^)	V_Meso_^d^ /(cm^3^·g^−1^)	Yield ^e^ /%	R_Meso_^f^
EC-10%	1160.34	25.61	0.40	0.20	53.16	50.7%
EC-15%	1206.30	26.08	0.62	0.38	50.49	61.1%
EC-25%	1418.77	27.76	0.76	0.71	48.52	93.9%
EC-35%	1099.09	28.79	0.61	0.53	47.10	86.4%
EC-40%	1182.57	28.41	0.59	0.50	47.23	85.1%
EC-45%	1023.89	28.90	0.53	0.44	45.41	83.0%
EC-50%	946.48	30.04	0.49	0.40	44.19	80.7%
EC-55%	888.95	31.68	0.44	0.31	40.48	71.1%
EC-1:2	1185.52	25.55	0.40	0.21	55.63	52.5%
EC-1:3	1367.31	26.73	0.46	0.34	50.49	74.8%
EC-1:4	1418.77	27.76	0.76	0.71	48.52	93.9%
EC-1:5	1099.09	27.46	0.61	0.53	47.39	86.9%
EC-2h	1418.77	27.76	0.75	0.70	60.72	93.0%
EC-4h	1613.28	26.21	0.82	0.78	53.14	95.1%
EC-6h	2033.87	26.66	1.11	1.07	43.88	96.4%
EC-8h	1048.17	26.63	0.63	0.55	40.92	87.3%
EC-350	1446.05	26.68	0.54	0.47	52.70	87.0%
EC-400	2033.87	26.66	1.11	1.07	43.88	96.4%
EC-450	1737.54	28.22	0.96	0.89	41.14	92.7%
EC-500	1553.15	28.06	0.89	0.82	39.53	92.1%

(^a^) Specific surface area was evaluated in the 0.05 < P·P_0_^−1^ < 0.1 pressure range; (^b^) average pore size of total pores; BJH Adsorption average pore diameter (4V/A). (^c^) Total pore volume calculated as the volume of the liquid at P·P_0_^−1^ ≈ 0.95. (^d^) Mesoporous volume, V_Meso_ = V_total_ − V_Micro_. (^e^) The yield of activated carbon was calculated based on the dry weight of EUO wood. (^f^) Distribution ratio of mesopore volume = Mesoporous volume/Total pore volume × 100%.

**Table 3:** The specific capacitance of ECs at 5 mV·s^−1^.

Sample	Specific Capacitance/(F·g^−1^)				Capacitance
	5/(mV·s^−1^)	10/(mV·s^−1^)	20/(mV·s^−1^)	50/(mV·s^−1^)	Retention/%
EC-10%	140	128	101	83	59%
EC-25%	168	159	142	102	61%
EC-40%	135	125	99	81	60%
EC-55%	116	101	87	66	57%
EC-1:2	75	63	55	41	54%
EC-1:3	140	127	100	81	58%
EC-1:4	168	159	142	102	61%
EC-1:5	82	76	60	53	64%
EC-2h	168	159	142	102	61%
EC-4h	208	196	160	129	62%
EC-6h	252	244	232	154	63%
EC-8h	193	171	154	127	66%
EC-350	175	153	138	105	60%
EC-400	252	244	232	154	63%
EC-450	210	186	148	130	62%
EC-500	198	177	134	115	58%

**Table 4:** Parameters for samples obtained from electrochemical impedance spectroscopy measurements.

Sample	*R_s_*^a^ (Ω)	*R_ct_*^b^ (Ω)	ESR ^c^ (Ω)
EC-10%	3.007	3.891	0.884
EC-25%	2.970	3.330	0.360
EC-40%	3.192	3.892	0.700
EC-55%	3.148	3.778	0.630
EC-1:2	2.738	4.103	1.365
EC-1:3	3.224	4.379	1.155
EC-1:4	3.004	3.309	0.305
EC-1:5	3.265	4.206	0.941
EC-2h	2.970	3.330	0.360
EC-4h	2.906	2.971	0.065
EC-6h	2.246	3.052	0.806
EC-8h	1.890	2.790	0.900
EC-350	2.782	2.823	0.050
EC-400	2.258	3.050	0.792
EC-450	2.343	2.488	0.145
EC-500	1.894	2.769	0.875

^a^ *R_s_* the intrinsic resistance of the substrate or electrolyte resistance. ^b^ *R_ct_* the charge transfer resistance. ^c^ ESR equivalent series resistance.

**Table 5:** The operating costs are based on units of specific capacity.

	EC-10%	EC-25%	EC-40%	EC-55%	EC-1:2	EC-1:3	EC-1:4	EC-1:5	EC-2h	EC-4h	EC-6h	EC-8h	EC-350	EC-400	EC-450	EC-500
EUO/CNY	2.00 × 10^−3^	2.00 × 10^−3^	2.00 × 10^−3^	2.00 × 10^−3^	2.00 × 10^−3^	2.00 × 10^−3^	2.00 × 10^−3^	2.00 × 10^−3^	2.00 × 10^−3^	2.00 × 10^−3^	2.00 × 10^−3^	2.00 × 10^−3^	2.00 × 10^−3^	2.00 × 10^−3^	2.00 × 10^−3^	2.00 × 10^−3^
Deionized water/CNY	1.20 × 10^−2^	1.20 × 10^−2^	1.20 × 10^−2^	1.20 × 10^−2^	1.20 × 10^−2^	1.20 × 10^−2^	1.20 × 10^−2^	1.20 × 10^−2^	1.20 × 10^−2^	1.20 × 10^−2^	1.20 × 10^−2^	1.20 × 10^−2^	1.20 × 10^−2^	1.20 × 10^−2^	1.20 × 10^−2^	1.20 × 10^−2^
Electricity for hydrothermal reaction/CNY	2.00	2.00	2.00	2.00	2.00	2.00	2.00	2.00	2.00	2.00	2.00	2.00	2.00	2.00	2.00	2.00
H_3_PO_4_/CNY	2.30 × 10^−5^	5.76 × 10^−5^	9.24 × 10^−5^	1.27 × 10^−4^	2.88 × 10^−5^	4.32 × 10^−5^	5.76 × 10^−5^	7.20 × 10^−5^	5.76 × 10^−5^	5.76 × 10^−5^	5.76 × 10^−5^	5.76 × 10^−5^	5.76 × 10^−5^	5.76 × 10^−5^	5.76 × 10^−5^	5.76 × 10^−5^
Electricity for drying/CNY	8.34	8.34	8.34	8.34	8.34	8.34	8.34	8.34	8.34	8.34	8.34	8.34	8.34	8.34	8.34	8.34
Electricity for activation/CNY	4.00	4.00	4.00	4.00	4.00	4.00	4.00	4.00	4.00	8.00	1.20 × 10^1^	1.60 × 10^1^	4.00	4.00	4.00	4.00
Hydrochloric acid/CNY	3.20 × 10^−2^	3.20 × 10^−2^	3.20 × 10^−2^	3.20 × 10^−2^	3.20 × 10^−2^	3.20 × 10^−2^	3.20 × 10^−2^	3.20 × 10^−2^	3.20 × 10^−2^	3.20 × 10^−2^	3.20 × 10^−2^	3.20 × 10^−2^	3.20 × 10^−2^	3.20 × 10^−2^	3.20 × 10^−2^	3.20 × 10^−2^
Electricity for heating/CNY	4.58 × 10^−1^	4.58 × 10^−1^	4.58 × 10^−1^	4.58 × 10^−1^	4.58 × 10^−1^	4.58 × 10^−1^	4.58 × 10^−1^	4.58 × 10^−1^	4.58 × 10^−1^	4.58 × 10^−1^	4.58 × 10^−1^	4.58 × 10^−1^	4.58 × 10^−1^	4.58 × 10^−1^	4.58 × 10^−1^	4.58 × 10^−1^
Wash Water/CNY	6.00 × 10^−1^	6.00 × 10^−1^	6.00 × 10^−1^	6.00 × 10^−1^	6.00 × 10^−1^	6.00 × 10^−1^	6.00 × 10^−1^	6.00 × 10^−1^	6.00 × 10^−1^	6.00 × 10^−1^	6.00 × 10^−1^	6.00 × 10^−1^	6.00 × 10^−1^	6.00 × 10^−1^	6.00 × 10^−1^	6.00 × 10^−1^
Carbon black/CNY	2.61 × 10^−3^	2.61 × 10^−3^	2.61 × 10^−3^	2.61 × 10^−3^	2.61 × 10^−3^	2.61 × 10^−3^	2.61 × 10^−3^	2.61 × 10^−3^	2.61 × 10^−3^	2.61 × 10^−3^	2.61 × 10^−3^	2.61 × 10^−3^	2.61 × 10^−3^	2.61 × 10^−3^	2.61 × 10^−3^	2.61 × 10^−3^
PTFE/CNY	1.31 × 10^−3^	1.31 × 10^−3^	1.31 × 10^−3^	1.31 × 10^−3^	1.31 × 10^−3^	1.31 × 10^−3^	1.31 × 10^−3^	1.31 × 10^−3^	1.31 × 10^−3^	1.31 × 10^−3^	1.31 × 10^−3^	1.31 × 10^−3^	1.31 × 10^−3^	1.31 × 10^−3^	1.31 × 10^−3^	1.31 × 10^−3^
SUM/CNY	1.54	1.54	1.54	1.54	1.54	1.54	1.54	1.54	1.54	1.54	1.54	1.54	1.54	1.54	1.54	1.54
The operating costs/(F·g^−1^·CNY-1)	1.10 × 10^−1^	9.20 × 10^−2^	1.14 × 10^−1^	1.33 × 10^−1^	2.06 × 10^−1^	1.10 × 10^−1^	9.20 × 10^−2^	1.88 × 10^−1^	9.20 × 10^−2^	9.40 × 10^−2^	9.30 × 10^−2^	1.42 × 10^−1^	8.80 × 10^−2^	6.10 × 10^−2^	7.40 × 10^−2^	7.80 × 10^−2^

## Data Availability

The data presented in this study are available in the article and can be shared upon request.

## Supplementary Materials

The following supporting information can be downloaded at: https://www.mdpi.com/article/10.3390/polym15030663/s1, Figure S1: (a): TGA and DTG of the EUO-wood; (b) TGA and DTG of the EUO-wood infiltrated by phosphoric acid; Figure S2: TEM images of EC-400 and EC-10%. TEM images of EC-400 and EC-10%; Figure S3: The CV curves of ECs at different scanning rates (phosphoric acid concentration and solid-liquid ratio); Figure S4: The CV curves of ECs at different scanning rates (activation time and activation temperature); Figure S5: Cyclic stability recorded for EC-10% and EC-400; Table S1: The specific capacitance of ECs at different current density; Table S2: Comparation of activated carbons prepared from different biomass for Supercapacitor application. References [46,47,48,49,50,51,52,53,54] are cited in the supplementary materials.
