# Supplementary material for: Controllable Preparation of Eucommia Wood-Derived Mesoporous Activated Carbon as Electrode Materials for Supercapacitors
Source: Polymers (Basel). 2023 Jan 28;15(3):663. doi: 10.3390/polym15030663 (PMC9920536; doi:10.3390/polym15030663)
Supplement: Supplementary file 1 [file polymers-15-00663-s001.zip › polymers-2180113-supplementary.pdf]

Supporting Information

Controllable Preparation of Eucommia Wood-Derived Mesoporous Activated Carbon as Electrode Materials for Supercapacitors

Hongyu Su [1,2], Caining Lan [1,2], Zhouping Wang [3], Lin Zhu [1,2] and Mingqiang Zhu [1,2,*]

1 College of Mechanical and Electronic Engineering, Northwest A&F University, Yangling 712100, China
2 Northwest Research Center of Rural Renewable Energy Exploitation and Utilization of M.O.A, Northwest A&F University, Yangling 712100, China
3 Horticultural Technology Station of Shaanxi, Xi'an 710003, China
* Correspondence: zmqsx@nwsuaf.edu.cn; Fax: +86-29-87092269

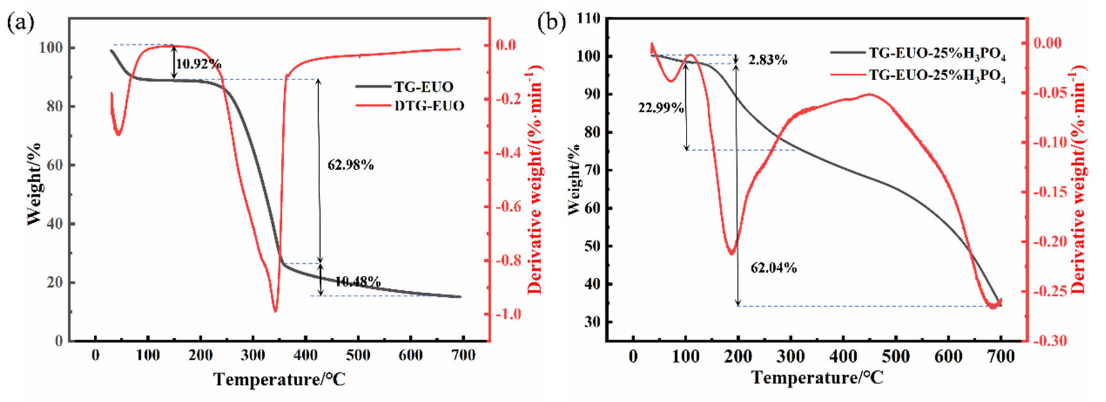

Figure S1. (a): TGA and DTG of the EUO-wood; (b) TGA and DTG of the EUO-wood infiltrated by phosphoric acid.

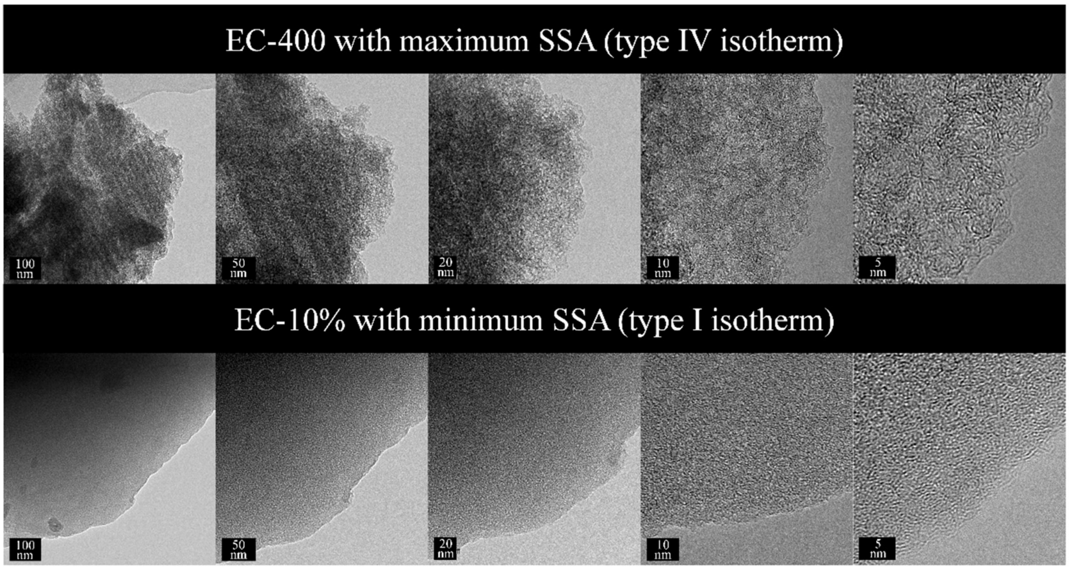

Figure S2. TEM images of EC-400 and EC-10%.

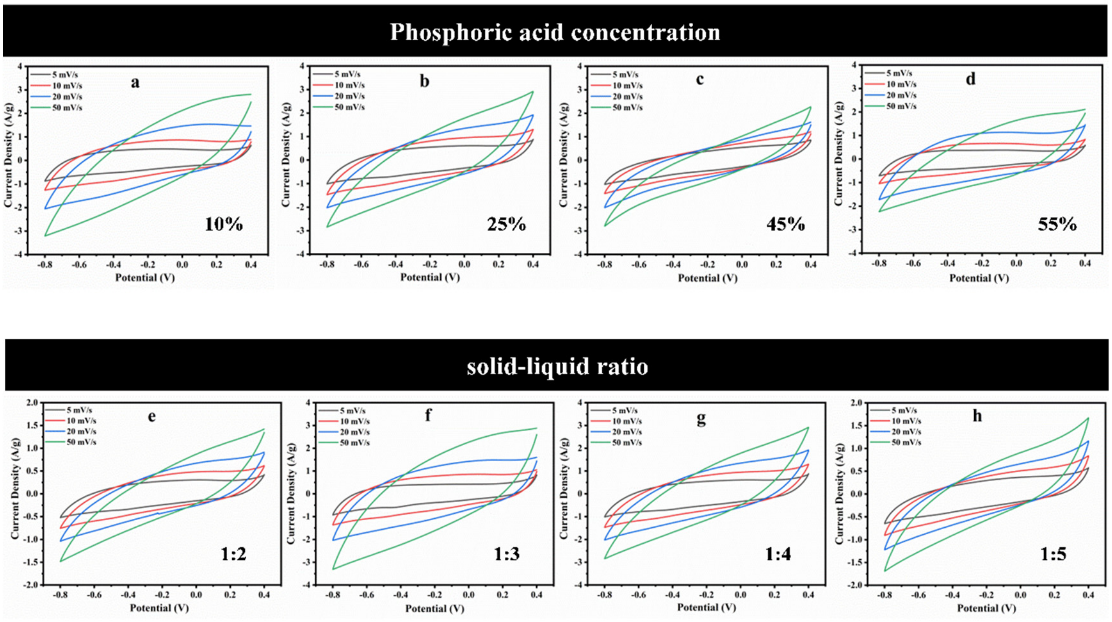

Figure S3. The CV curves of ECs at different scanning rates (phosphoric acid concentration and solid-liquid ratio).

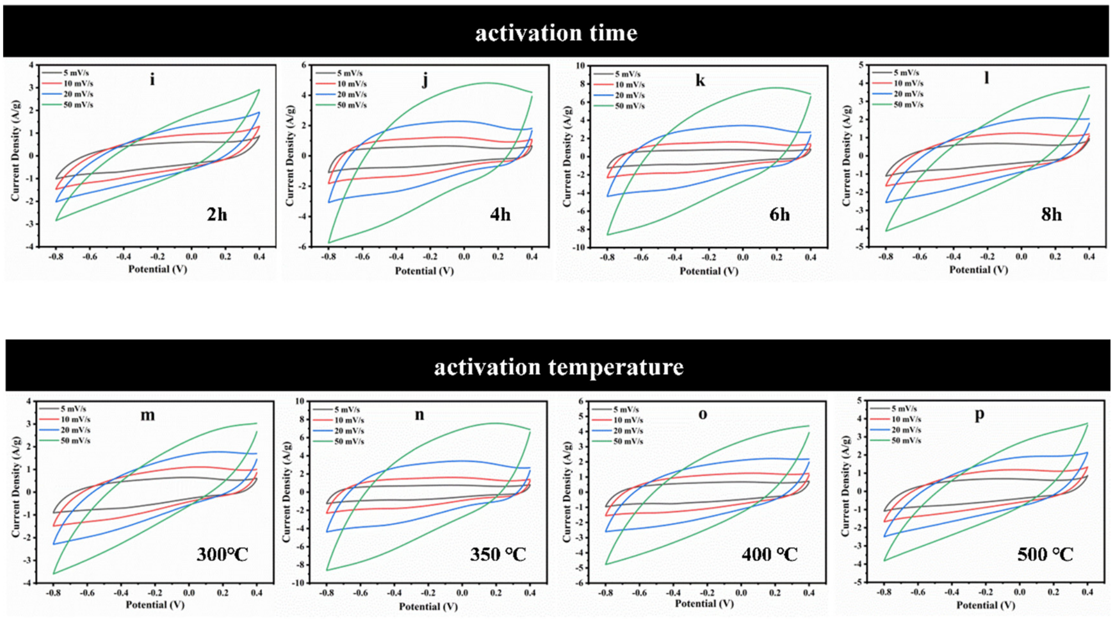

Figure S4. The CV curves of ECs at different scanning rates (activation time and activation temperature).

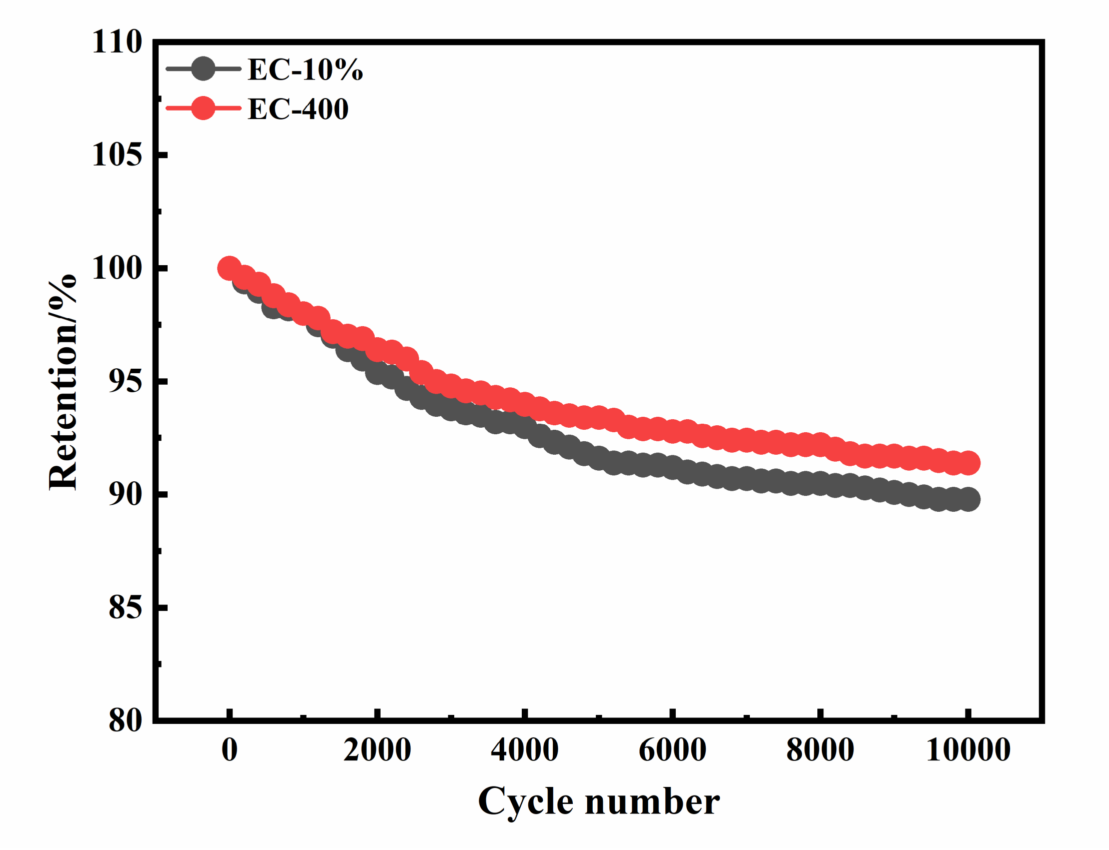

Figure S5. Cyclic stability recorded for EC-10% and EC-400.

Table S1: The specific capacitance of ECs at different current density.

Sample	Specific capacitance/(F • g^{-1})				Capacitance Retention/%
	0.2/(A·g^{-1})	0.5/(A·g^{-1})	0.8/(A·g^{-1})	1.2/(A·g^{-1})	
EC-10%	153.7	148.0	134.9	112.8	73.4%
EC-25%	194.6	181.1	166.4	148.5	76.3%
EC-40%	178.4	169.3	145.5	135.0	75.7%
EC-55%	171.1	157.9	144.6	134.1	78.4%
EC-1:2	148.3	125.2	109.9	101.3	68.3%
EC-1:3	177.5	167.0	150.1	135.4	76.2%
EC-1:4	194.6	181.1	166.4	139.9	76.3%
EC-1:5	171.4	160.6	140.0	126.0	73.5%
EC-2h	194.6	181.1	166.4	139.9	76.3%
EC-4h	226.7	211.4	185.3	177.3	78.2%
EC-6h	257.8	247.5	220.2	208.7	81.0%
EC-8h	171.7	161.3	155.4	141.3	82.3%
EC-350	186.5	171.0	164.4	147.5	79.1%
EC-400	257.8	247.5	220.2	208.7	81.0%
EC-450	238.2	227.7	210.0	198.7	83.4%
EC-500	210.0	194.3	185.5	171.6	81.7%

Table S2 Comparation of activated carbons prepared from different biomass for Supercapacitor application

Starting materials	Activation condition		Physical Characteristics		Electrochemical Characteristics			Electrolyte	Ref
	a	b (°C)	S_{BET} (m^2/g)	c (cm^3/g)	d (Fg^{-1})	e (Ag^{-1})	f		
Soya Beans	KOH	700	1208	-	149	0.5	89.3% in 5000	KOH/PVA	Chen et al. 2019 [1]
Binata	KOH	852	2273	1.19	373	0.5	94.6% in 10000	6 M KOH	Liu et al. 2016 [2]
Tree bark	KOH	800	1018	0.67	191	1	100% in 5000	Na_2SO_4	Momodu et al. 2017 [3]
Brussel	KOH	900	2267	1.32	255	0.5	99.55% in 5000	6 M KOH	Li et al. 2016 [4]
Rotten carrot	$ZnCl_2$	700	1253	0.95	137	1	-	6 M KOH	Ahmed et al. 2018 [5]
Cottonseed meal	K_2CO_3	800	2361	1.41	313.4	0.1	86.4% in 20000	6 M KOH	Jia et al. 2022 [6]
Tamarindus indica Fruit Shell	H_3PO_4	900	846	0.556	287	1	92.5% in 5000	1 M KOH	Sivachidambaram et al. 2018 [7]
Baobab shell	H_3PO_4	800	911	0.47	355	1	99.7% in 5000	4 M KOH	Mohammed et al. 2019 [8]
Olive residue	H_3PO_4	840	1390	0.61	150	0.25	91% in 12500	1 M Na_2SO_4	Ma 2017 [9]
EUO (EC-400)	H_3PO_4	400	2033.87	1.11	252	0.2	91.4% in 10000	1 M H_2SO_4	This study

a) Activation agent, b) Activation temperature, S_{BET}) Brunauer-Emmett-Teller surface area, c) Total pore volume, d) Specific capacitance, e) Current density, f) Stabili

References

1. Chen, M.; Yu, D.; Zheng, X.; Dong, X. Biomass based N-doped hierarchical porous carbon nanosheets for all-solid-state supercapacitors. *J. Energy Storage* **2019**, 21, 105-112.
2. Liu, B.; Chen, H.; Gao, Y.; Li, H. Preparation and capacitive performance of porous carbon materials derived from eulaliopsis binata. *Electrochem. Acta* **2016**, 189, 93-100.
3. Momodu, D.; Madito, M.; Barzegar, F.; Bello, A.; Khaleed, A.; Olaniyan, O.; Dangbegnon, J. Manyala, N. Activated carbon derived from tree bark biomass with promising material properties for supercapacitors. *J. Solid State Electrochem.* **2017**, 21, 859-872.
4. Li, J.; Zan, G.; Wu, Q. Nitrogen and sulfur self-doped porous carbon from brussel sprouts as electrode materials for high stable supercapacitors. *RSC Adv.* **2016**, 6, 57464-57472.
5. Ahmed, S.; Ahmed, A.; Rafat, M. Supercapacitor performance of activated carbon derived from rotten carrot in aqueous, organic and ionic liquid based electrolytes. *J. Saudi Chem. Soc.* **2018**, 22, 993-1002.
6. Jia, B.; Mian, Q.; Wu, D.; Wang, T. Heteroatoms self-doped porous carbon from cottonseed meal using K_2CO_3 as activator and DES electrolyte for supercapacitor with high energy density. *Materials Today Chem.* **2022**, 24.
7. Sivachidambaram, M.; Vijaya, J.J.; Niketha, K.; Kennedy, L.J.; Elanthamilan, E.; Merlin, J.P. Electrochemical studies on tamarindus indica fruit shell bio-waste derived nanoporous activated carbons for supercapacitor applications. *J. Nanosci.* **2019**, 19(6), 3388-3397.
8. Mohammed, A.A.; Chen, C.; Zhu, Z. Low-cost, high-performance supercapacitor based on activated carbon electrode materials derived from baobab fruit shells. *J. Colloid Interface Sci.* **2019**, 538, 308-319.
9. Ma Y: Comparison of Activated Carbons Prepared from Wheat Straw via $ZnCl_2$ and KOH Activation. *Waste Biomass Valorization* **2016**, 8, 549-559.
